# Homologous alignment cloning: a rapid, flexible and highly efficient general molecular cloning method

**DOI:** 10.7717/peerj.5146

**Published:** 2018-06-29

**Authors:** Lendl Tan, Emily J. Strong, Kyra Woods, Nicholas P. West

**Affiliations:** School of Chemistry and Molecular Biosciences, Australian Infectious Diseases Research Centre, University of Queensland, Brisbane, QLD, Australia

**Keywords:** Molecular cloning, Low cost, High efficiency, Simple, Ligation-independant, High fidelity, Rapid, Homologous alignment cloning, HAC

## Abstract

Homologous alignment cloning (HAC) is a rapid method of molecular cloning that facilitates low-cost, highly efficient cloning of polymerase chain reaction products into any plasmid vector in approximately 2 min. HAC facilitates insert integration due to a sequence alignment strategy, by way of short, vector-specific homology tails appended to insert during amplification. Simultaneous exposure of single-stranded fragment ends, utilising the 3′→5′ exonuclease activity of T4 DNA polymerase, creates overlapping homologous DNA on each molecule. The exonuclease activity of T4 polymerase is quenched simply by the addition of EDTA and a simple annealing step ensures high yield and high fidelity vector formation. The resultant recombinant plasmids are transformed into standard *E. coli* cloning strains and screened via established methods as necessary. HAC exploits reagents commonly found in molecular research laboratories and achieves efficiencies that exceed conventional cloning methods, including another ligation-independent method we tested. HAC is also suitable for combining multiple fragments in a single reaction, thus extending its flexibility.

## Introduction

Molecular cloning is a process central to modern molecular biology that was first launched by the discovery of restriction endonucleases (RE) and their ability to recognise and cleave specific DNA sequences (Nathans & Smith, 1975). One of the most basic and commonly used methods of cloning involves the insertion of a fragment of interest (often a coding gene) into a plasmid vector (Helinski, 1977). The utility and benefits of molecular cloning are well-known, with a myriad of potential outcomes, including the ability to express a gene of interest in diverse backgrounds to characterise phenotypic consequences of specific genes (Nasmyth, 1978; Struhl & Davis, 1977; Wood et al., 1983), virulence determinants (Macrina, 1984) and for the expression and purification of recombinant proteins for a wide-array of applications (Bell et al., 2013; Celie, Parret & Perrakis, 2016; Derewenda, 2004; Joosten et al., 2003; Koth & Payandeh, 2009), to name just a few.

Conventional cloning methods involve the linearisation of the vector backbone with an RE at one or more sites followed by subsequent DNA ligase-facilitated ligation of vector and insert DNA which has been modified in the same way (Hamer & Thomas, 1976). While conventional cloning is a tried and tested method that remains widely used, there are often significant limitations, including the propensity for vector self-ligation or the requirement of directional cloning that can impede the efficiency, or simply the often-encountered practical limitation of available RE sites unique to the vector but not the insert. Over the years many different ways to circumvent issues and improve the efficiency of conventional cloning have been proposed (Lessard, 2013), regardless, many of these modifications have brought limited efficiency improvements despite decades of method development (Matsumura, 2015).

Ligation-independent cloning (LIC) methods are alternatives to conventional cloning that do not require the use of DNA ligase. Instead, these methods often rely on the T4 DNA polymerase (T4 pol), an enzyme that also exhibits a 3′→5′ exonuclease activity in the absence of dNTPs (Englund, 1971). An early LIC–polymerase chain reaction (PCR) method describes the usage of T4 pol in cloning, where both insert and vector are PCR amplified with 13 nucleotide homology overhang sequences lacking dCMP, thereby allowing treatment with T4 pol in the presence of dGTP to generate single-stranded homology sequences to mediate circularisation (Aslanidis & de Jong, 1990). While this methodology represented a new generation of cloning techniques, the development and subsequent requirement of specific vector sequences and purpose-built LIC vectors limits its flexibility and utility. This limitation saw the need for the production of many task-specific vectors, such as those incorporating fusion tags and/or vectors for protein purification studies (Eschenfeldt et al., 2009; Stols et al., 2002).

A number of other LIC methods have since been proposed, and these include the sequence and ligation-independent cloning (SLIC) (Li & Elledge, 2007), polymerase incomplete primer extension (PIPE) cloning (Klock et al., 2008), overlap extension cloning (OEC) (Bryksin & Matsumura, 2010), FastCloning (Li et al., 2011), quick and clean cloning (Thieme et al., 2011), exonuclease and ligation-independent cloning (ELIC) (Koskela & Frey, 2015), and one-step SLIC (Islam et al., 2017; Jeong et al., 2012). Among these methods, the SLIC, PIPE and OEC have been tested and discussed in an independent study raising their merits and limitations (Stevenson et al., 2013). While ELIC claims to be the fastest of all LIC methods, the reliability of this procedure has been called into question (Islam et al., 2017).

Here, we present a modified LIC method known as homologous alignment cloning (HAC). HAC is a straight forward, low cost, and rapid method for directional cloning of PCR products. We demonstrate here efficiency yields, for single fragment cloning, of virtually 100% of all resultant colonies. Furthermore, HAC utilises reagents commonly used in molecular research labs. We present a detailed description of the HAC method along with evidence of optimised efficiency. For purposes of demonstration, we report here directly observable phenotypes via cloning the green fluorescence protein (*gfp*) gene, or chloramphenicol and kanamycin resistance cassettes.

## Materials and Methods

### Bacterial strains and plasmids used in this study

All cloning was performed using the prototypical *E. coli* cloning strain, DH5α. Bacteria were grown in Luria-Bertani (LB) broth or on LB agar supplemented with Ampicillin at 100 μg/ml, Kanamycin at 50 μg/ml, chloramphenicol at 25 μg/ml. The pUC19 plasmid (Norrander, Kempe & Messing, 1983) was used in all cloning experiments performed in this study. All oligonucleotides used in this study were synthesised by Sigma-Aldrich. Primers were designed to amplify the insert of interest along with appropriate overhangs homologous to the pUC19 vector at the *Hin*dIII site (HAC tails) (refer to results for detailed description of HAC tail design). Table S1 lists the primers used and the plasmids constructed in this study.

### General DNA manipulation and genetic techniques

Polymerase chain reaction was performed using the Q5^®^ High-Fidelity DNA Polymerase according to manufacturer’s recommended cycling conditions (New England Biolabs, Ipswich, MA, USA). Plasmid DNA was isolated using the QIAprep Spin Miniprep kit (Qiagen, Hilden, Germany). Restriction endonuclease *Hin*dIII was used according the manufacturer’s recommendation (New England Biolabs, Ipswich, MA, USA). PCR products and other DNA fragments were visualised using agarose gel electrophoresis and purified using the QIAquick PCR Purification kit (Qiagen, Hilden, Germany). Purified plasmid and DNA fragments were quantified using the Nano-drop 2000 Spectrophotometer (Thermo Fisher Scientific, Waltham, MA, USA).

### Determination of the T4 pol exonuclease activity rate

A total of 30 reactions (20 μl total volume) containing 100 ng of purified *gfp* PCR product, 0.2 μl (1 U) T4 pol (Thermo Fisher Scientific, Waltham, MA, USA) and 1X reaction buffer each were prepared on ice in a 96-well PCR plate. The first reaction representing *T* = 0 was prepared with 1 μl of 0.5 M EDTA already added to the well to serve as the negative control (no activity). To initiate the assay, the PCR plate was transferred to a PCR dry-block incubator at 37 °C and 1 μl of 0.5 M EDTA was subsequently added and mixed into each well at regular intervals for approximately 7 min, followed by an 8-min time point, and each 2 min thereafter for an additional 10 min. A total of 20 μl of 2X Sybr Green I was added to each reaction and transferred to a flat-bottom 96-well plate for analysis. Relative fluorescence of Sybr stained PCR product was also assessed by preparing five different double-stranded to single-stranded ratios (100:0, 75:25, 50:50, 25:75, 0:100) with 100 ng of total DNA in each. The single-stranded PCR product was prepared by heating at 98 °C for 15 min before rapidly cooling on ice. The relative fluorescence of each reaction was determined using the FLUOstar Omega (BMG Labtech, Offenburg, Germany) plate reader at an excitation/emission wavelength of 480/520 nm. As an additional test, 100 μl of Hyperladder^™^ 100 bp (Bioline, London, UK) was purified using the QIAquick PCR Purification kit and used to set up five reactions (20 μl total volume) containing 5 μl of purified ladder (650 ng total DNA), 0.4 μl (2 U) of T4 pol and 1X reaction buffer. The first reaction was prepared with 1 μl 0.5 M EDTA. The remaining four tubes were incubated at 37°C before being sequentially stopped by the addition of 1 μl 0.5 M EDTA at the 30 s, 1 min, 3 min and 5 min time point. Each reaction was purified once more and the entire contents separated on a 2% agarose gel.

### Homologous alignment cloning

A 20 μl HAC reaction containing 50 ng of linearised vector DNA, an appropriate quantity of insert DNA at a 3:1 insert:vector molar ratio, 0.2 μl (1 U) T4 pol and 1X reaction buffer was prepared on ice. The reaction was incubated at 37 °C for 1 min and stopped by the addition of 1 μl of 0.5 M EDTA. To reduce mismatch annealing, the reaction was incubated at 60 °C for 1 min, removed from heat and allowed to cool slowly to ambient temperature (annealing step). The resulting reaction was then transformed into chemically competent DH5α. An alternative HAC procedure which utilised dNTPs to reverse T4 pol exonuclease activity was tested instead of EDTA. Upon the incubation of the reaction at 37 °C for 1 min, the reaction was immediately allowed to anneal at 60 °C for 1 min and cooled to ambient temperature slowly. A final concentration of 0.1 mM of each dNTPs was then added to the reaction and incubated at 12 °C for 30 min to allow for the repairing of the single-stranded gaps between the annealed insert-vector. The reaction was then transformed into DH5α.

### Preparation of competent cells

Competent DH5α *E. coli* was prepared using the TSS buffer method (Chung, Niemela & Miller, 1989). Briefly, DH5α was grown in 100 ml of LB broth, shaking at 37 °C to an optical density at 600 nm (OD_600_) of 0.6. The culture was chilled on ice and cells were pelleted via centrifugation at 4 °C. The cell pellet was then resuspended in 5 ml TSS buffer (10% PEG 3350, 50 mM MgSO4, 5% DMSO in LB medium) and transferred into 100 μl aliquots, snap frozen in liquid nitrogen and stored at −80 °C. All cloning experiments in this study were performed using the same batch of competent cells. Prior to use, aliquots of competent cells were first thawed on ice.

### Bacterial transformation

A total of 10 μl of the HAC reaction mix was added to 100 μl of competent cells and incubated on ice for 30 min. The cells were then subjected to heat shock at 42 °C for 90 s and chilled on ice for 90 s. Cells were recovered in 1 ml of LB broth for 1 h at 37 °C and plated on LB agar plates supplemented with the appropriate antibiotics when necessary. Colonies containing pUC19-*gfp* clones were visualised using the Amersham Imager 600 (GE Healthcare, Chicago, IL, USA) to identify and quantify fluorescent colonies.

## Results

The central principle of HAC is based on the annealing of DNA fragments (inserts) with vector homologous sequence, at the molecule ends. This homology is generated on the insert, to match that of the target vector sequence, adjacent to any restriction site of choice. The vector specific sequence is appended onto the insert during PCR amplification utilising tailed primers (HAC-tails). In a single reaction, the insert and RE-digested vector are treated with T4 pol, producing single stranded, homologous ends which are subsequently annealed before being transformed into a standard, competent cloning *E. coli* strain.

### Primer HAC-tail design requirements

**Figure 1:**
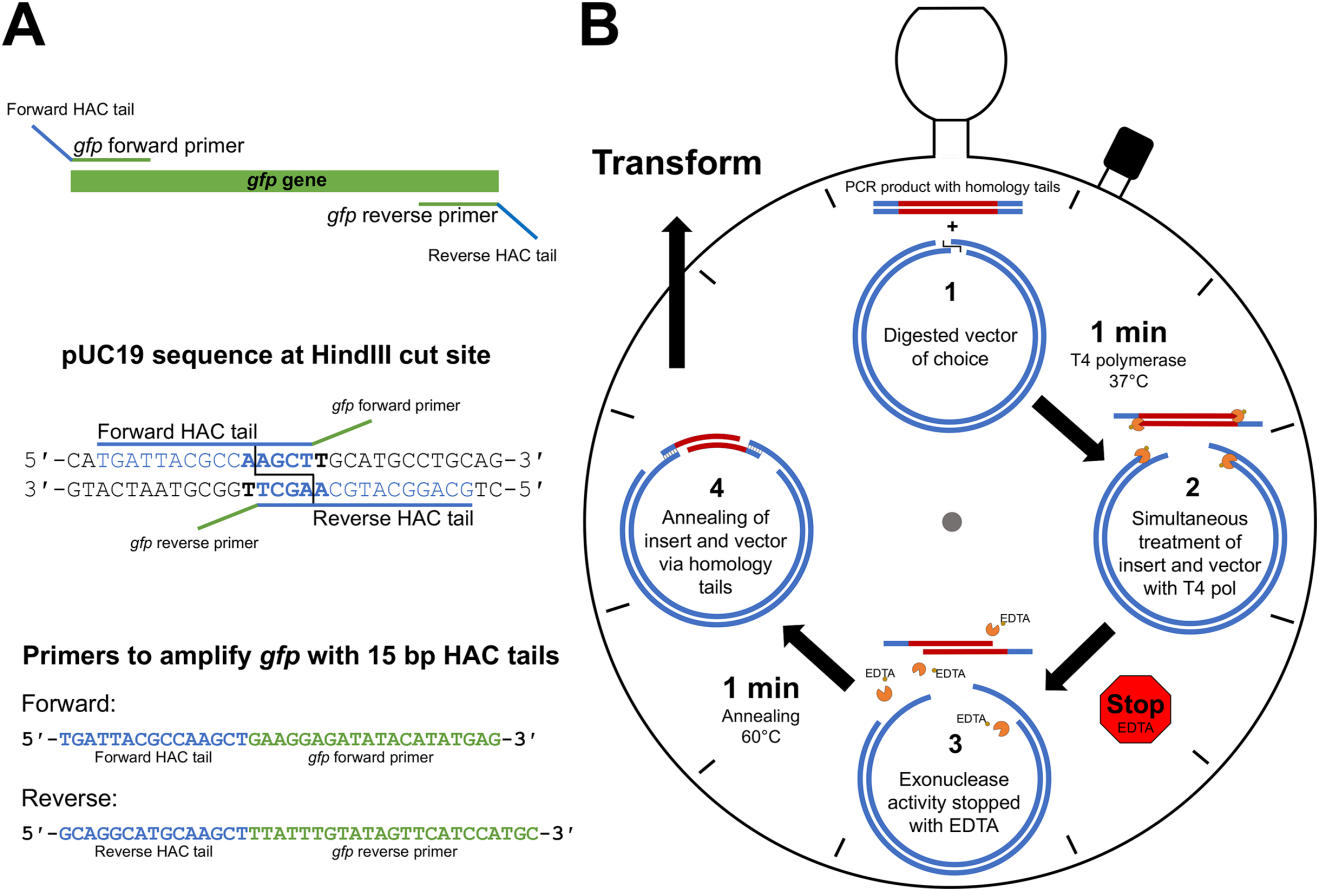
HAC: primer design and workflow. (A) An example primer design strategy used in this study where the *gfp* gene was amplified with primers appended with 15 bp HAC-tails specific for pUC19/*Hin*dIII. (B) Step-by-step schematic describing the method for the 2 min-HAC.

The first step of HAC involves primer design that incorporates vector-specific HAC tails onto the amplified PCR product, thereby allowing the insert to anneal to the linearised vector. The HAC primer tail is dependent on the sequence adjacent to the selected vector RE cut site, taking into account the bases that will remain following the 3′→5′ exonuclease action of T4 pol, irrespective of RE cleavage pattern. On the 5′ end of the forward PCR primer, sequence complementary to the vector negative strand is selected and incorporated. Conversely, sequence complementary to the positive strand on the vector is incorporated into the 5′ end of the reverse PCR primer. Figure 1A depicts the HAC primer design strategy, illustrating the approach used for the incorporation of *gfp* into pUC19 at the *Hin*dIII cut site.

### Outline of procedure

The desired insert is first PCR amplified utilising the HAC-tailed primers while the desired vector is digested with a restriction enzyme of choice. The PCR product is then purified with a suitable DNA clean-up kit. Linearised vector and insert are made single stranded at their ends through the 3′→5′ exonuclease action of T4 pol. The reliability of the DNA clean-up kit for the removal of unincorporated dNTPs following PCR is crucial, which if remain in the reaction will nullify the T4 pol exonuclease activity. A single tube reaction (Fig. 1B) is set-up with digested vector of choice, purified PCR product (insert), T4 pol buffer and T4 pol. We have tested insert to vector ratios of up to 10:1 and found that a 3:1 molar ratio yielded outstanding results. The reaction is incubated for 1 min at 37 °C, after which the reaction is stopped by the addition of EDTA, thereby generating insert and vector molecules with 5′- single-stranded tails. To minimise mismatch nucleotide paring between the complementary strands of vector and insert, the reaction is incubated for 1 min at 60 °C, removed from heat and allowed to cool to room temperature, at which point strand annealing is complete and the resultant recombinant plasmid is ready for transformation (Fig. 1B). The reaction mix is then transformed into a chemically competent *E. coli* cloning strain via heat shock and the resultant transformants are screened by standard methods. Key steps of the procedure have been experimentally optimised, as described below, with outcomes being included in the final recommended method.

### Determination of the appropriate exonuclease treatment time

**Figure 2:**
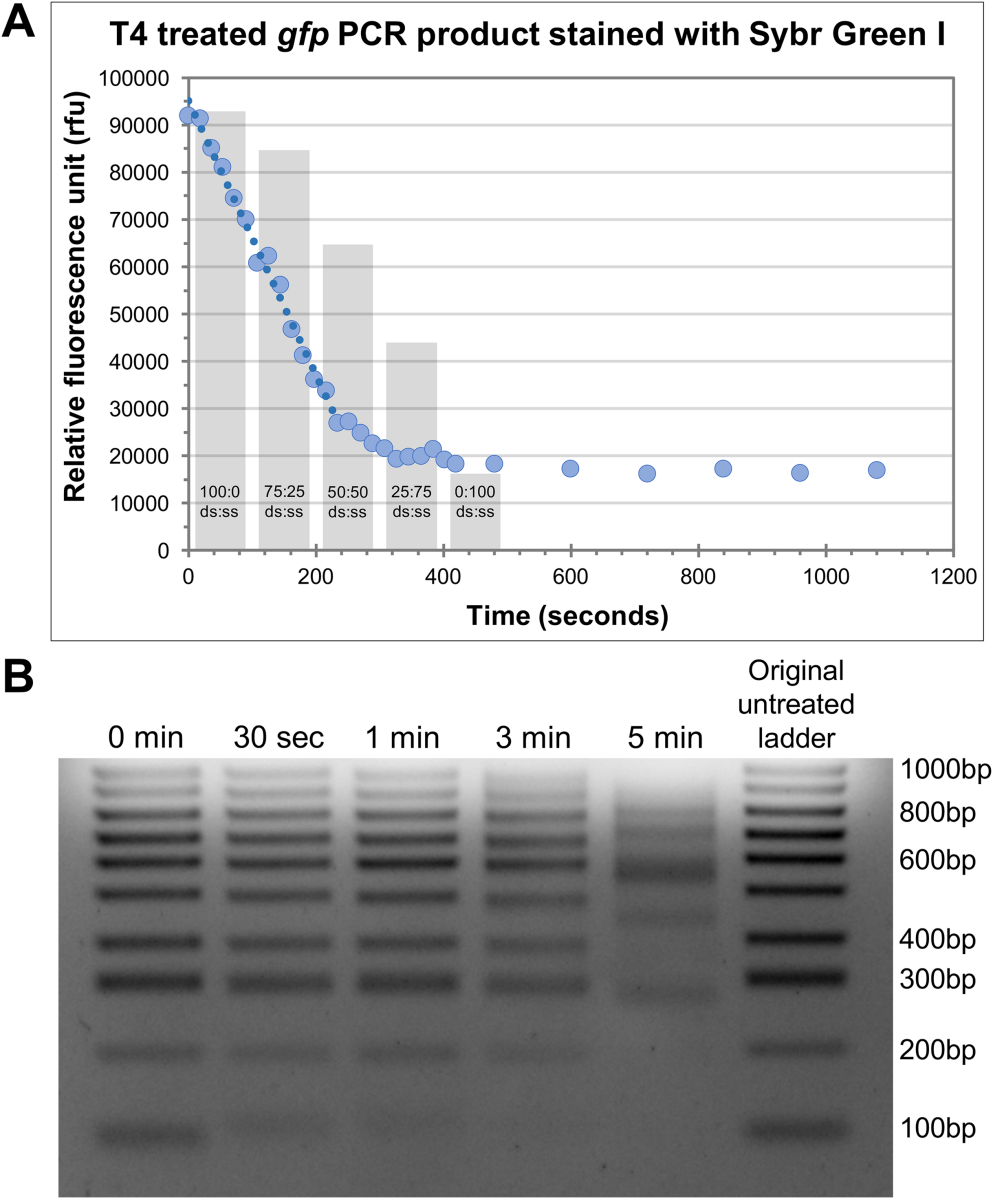
Defining the rate of exonuclease activity of T4 DNA polymerase. (A) Declining relative fluorescence units (rfu) during treatment of the *gfp* PCR product (762bp) with T4 DNA exonuclease. Multiple individual reactions were synchronously started and sequentially stopped at indicated time-points by the addition of EDTA. Sybr Green I was added to each reaction at its completion and samples were measured for Sybr Green fluorescence. A linear decrease in rfu is observed for approximately 4 min (240 s), highlighted by the trend line. Columns indicate the rfu of control double-stranded to single-stranded DNA samples containing the *gfp* PCR product at ratios indicated. (B) Gel electrophoresis of 100 bp ladder treated with T4 DNA exonuclease for 30 s, 1, 3 and 5 min. A decrease in low molecular weight band intensity was observed after just 30 s of treatment (100 bp). The 100 bp band was entirely degraded by 3 min. At 5 min, several larger bands began to show altered electrophoretic migration rates indicating significant fragment modification.

An important aspect of efficiency for HAC involved the determination of the optimal incubation time with T4 pol to generate the single-stranded overhangs on both the insert and vector. To achieve this optimally, it is necessary to understand the rate at which T4 pol exerts its exonuclease activity. Given too long there is a risk of the insert being completely degraded, whereas too short a treatment time may result in insufficient vector/insert being single stranded and not available to anneal. We assessed this in two ways, i.e. measurement of the ‘rate of decay’ of Sybr fluorescence, and, electrophoretic mobility shift. For Sybr fluorescence, we tested this by using 100 ng of a purified 762 bp PCR product (*gfp*) together with 1 U T4 pol (per reaction) and incubating the samples at 37 °C. At the desired time-points, individual reactions were stopped with the addition of EDTA and Sybr Green I, a double-stranded DNA (dsDNA) stain, was added. Due to the inability of Sybr Green I to effectively stain single stranded DNA, the relative quantity of dsDNA can be measured as relative fluorescence units (rfu) against time in seconds (Fig. 2A). We observed a reproducible linear decrease in rfu to approximately the 4-min time point (Fig. 2A). From there, the rate of fluorescence decline slows down to a near-plateau beyond the 5-min time point. It is likely that the linear section of the curve over the first 4 min represents a linear increase in single-strandedness. To assess the linearity of Sybr incorporation and resultant fluorescence during this analysis, we prepared 100 ng mixed samples of double-stranded and single-stranded DNA in defined ratios. The Sybr fluorescence of these control samples aligned very closely with that of our T4 treated DNA (Fig. 2A, grey columns), thereby validating our approach. A rudimentary rate of nucleotide removal can be calculated from the curve. Using 26,000 rfu (4-min time point) as an approximate indication of complete fragment degradation (initial size 762 bp), and 90,000 rfu (0 s time point) as the indication of the full-length fragment, we can consider a decrease of 64,000 rfu as the amount of signal lost through the exonuclease activity of T4 pol to degrade the entire PCR product. This translates to approximately 84 rfu/base. The rate of decrease for a reduction of 64,000 rfu over a 4-min time period equates to approximately 266 rfu/sec. Based on these values, we would calculate the rate of degradation to be approximately 3.2 bases/s. Considering that the T4 pol is exerting its exonuclease activity on both ends, the overall T4 pol exonuclease rate would be approximately half this figure, at 1.6 bases/s or 96 bases/min/molecule end.

To further appreciate the effects of T4 pol exonuclease activity on different sized products, we observed the effects of T4 pol together with a 100 bp ladder, treated for 30 s, 1 min, 3 min and 5 min. The outcomes were then visualised via gel electrophoresis (Fig. 2B). Even at 30 s, a shift in the 100 bp band indicates that it was modified by the treatment, resulting in altered electrophoretic properties likely due to the decreasing proportion of dsDNA modifying the molecular weight and charge of the fragment, and/or increased conformational flexibility due to the extended single-stranded regions. Treatment for 1 min has degraded a significant portion of the 100 bp fragment while at 3 min, it has been completely degraded. In addition, many of the larger bands also display altered migration rates at 5 min incubation. Based on these two results, we determined that 30 s incubation time is adequate for the generation of single-stranded tails, however we recommend 1 min at 37 °C for effective cloning, taking into account possible variation in rates and efficiency of the enzyme due to brand/source, or storage conditions; while ensuring that the insert of choice (even small inserts) remains protected from complete degradation.

### Optimisation of HAC tail lengths

**Figure 3:**
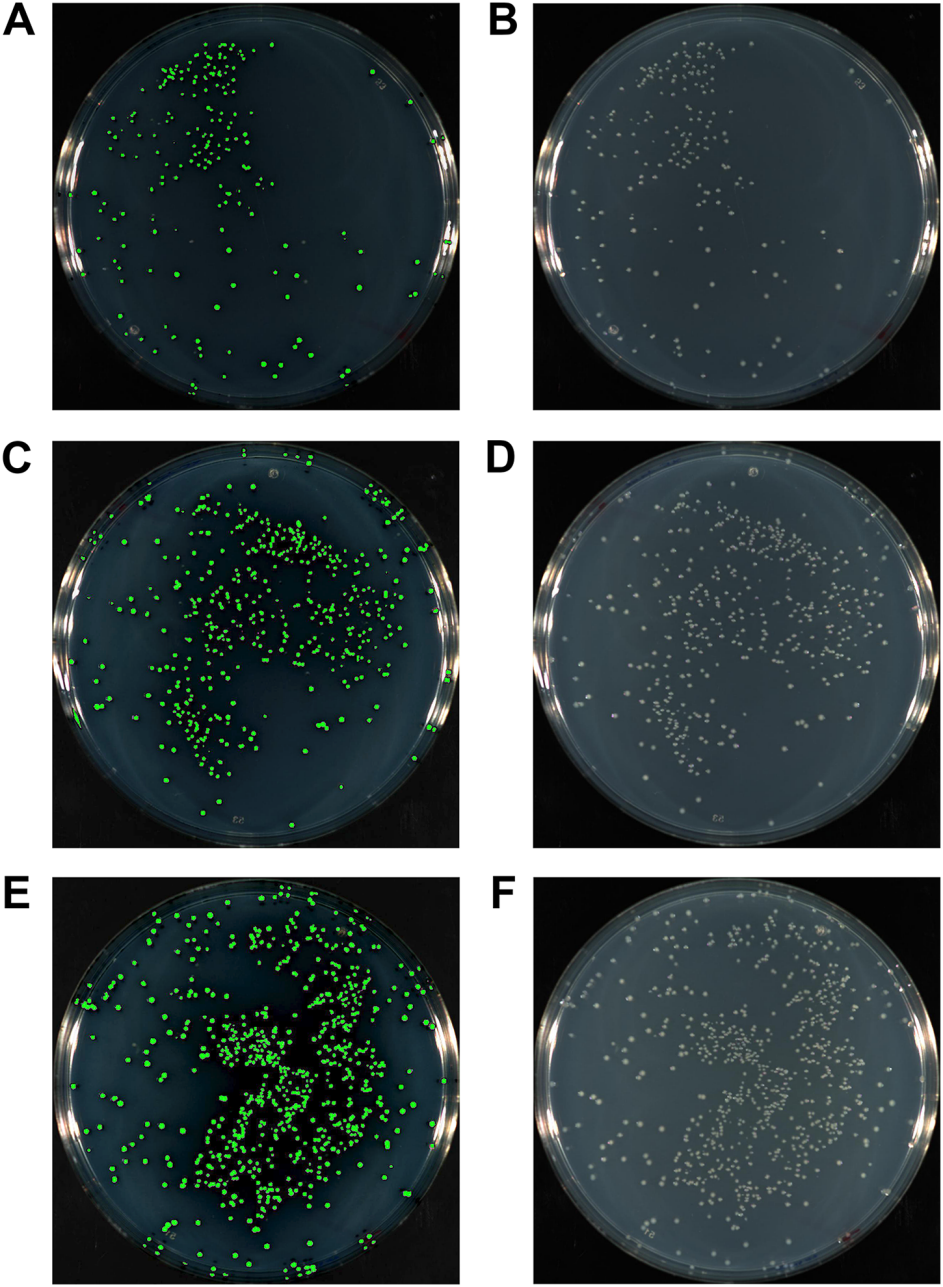
The gfp gene amplified with either 15 bp (A, B), 20 bp (C, D) or 25 bp HAC-tails (E, F) were cloned into pUC19 via HAC. 100 ml of each transformation post-recovery was plated on LB agar supplemented with Ampicillin. Assessment of colonies after 18 hr growth was performed with a photographic imager equipped with fluorescence detection. Images depicted were captured in both fluorescent (Cy3 filter) + colourimetric overlay (A, C, E) and colourimetric only (B, D, F) camera modes. Total numbers of fluorescing colonies as a percentage of total CFUs represent 96%, 98% and 99% for 15 bp, 20 bp and 25 bp HAC-tails respectively.

The complementary sequence of the HAC tails between the insert and vector is key for the efficient and specific annealing between the two, thereby allowing for highly successful cloning and cost minimisation. We compared the efficiency of HAC using inserts with 15, 20 or 25 bp HAC primer tails. At the same time, we also explored the potential benefit offered by T4 pol reversal to repair ssDNA gaps following fragment annealing. We cloned the *gfp* gene into pUC19, at a 3:1 insert to vector molar ratio using 50 ng of vector linearised at the *Hin*dIII site. The cloning of the *gfp* gene allowed for a simple and unbiased method to evaluate the success of the cloning procedure through identification of fluorescent colonies (Fig. 3). This strategy allows for immediate quantification of positive clones amongst all resultant colonies (Table 1). Cloning with 15 bp HAC-tails resulted in 226 colonies from plating 100 μl of transformants (out of 1 ml recovered volume; equivalent to 2.5 ng of vector DNA or ∼9 × 10^4^ cfu/μg of vector DNA), of which, 217 were fluorescent, which equates to 96% success rate of *gfp* insertion. An increase in the length of the HAC-tails increased the total number of transformants (467 or ∼1.9 × 10^5^ cfu/μg and 761 or ∼3 × 10^5^ cfu/μg colonies for 20 bp and 25 bp HAC tails, respectively), increasing overall success rate to 98% and 99%, respectively. As a reference, the assessed competency of the batch of cells used in this study was a modest 5 × 10^6^ cfu/μg of pUC19, further highlighting the efficiency of the method considering the modest level of competency of the cells used. Considering these exceptional cloning percentages, we recommend the design of 15 bp HAC-tails; using HAC tails beyond 15 bp would only serve to increase in the cost of primer synthesis, without an appreciable benefit.

**Table 1:** Efficiency of HAC for single insert cloning.

Cloning of *gfp* into pUC19						
	Stopped with EDTA			Repaired with dNTPs		
	Fluorescent colonies	Total colonies	% success	Fluorescent colonies	Total colonies	% success
15 bp overhangs	217	226	96	143	175	82
20 bp overhangs	458	467	98	367	415	88
25 bp overhangs	754	761	99	625	686	91

### Assessment of alternative exonuclease arresting method

T4 DNA polymerase will revert to its preferred 5′→3′ polymerase activity in the presence of dNTPs. Addition of dNTP to the reaction post insert annealing will facilitate plasmid ssDNA gap repair. This can be performed as an alternative to the addition of EDTA to reduce the possibilities of extended single-strandedness or unchecked exonuclease activity. We therefore tested and compared efficiencies of this alternative method and noticed no improvement in the efficiency when dNTP is used, indicating that this addition is unnecessary for the procedure (Table 1). We therefore recommend omitting this additional step in favour of the simpler and more cost effective EDTA stop solution.

### HAC for multiple fragment cloning

It is often desirable to produce vectors containing multiple inserts; to do so in a single reaction saves considerable time in the lab. We therefore next investigated the efficiency of utilising HAC for the simultaneous cloning of two inserts into a vector. To clearly demonstrate this, we amplified two antibiotic resistance cassettes carrying the *cat* and *neo* genes (*cm^r^* and *kan^r^*) conferring resistance to chloramphenicol (Cm) and kanmamycin (Kan), respectively. Appropriate HAC tails (15, 20, 25 bp) were designed for the junction between the *cm^r^* and *kan^r^* cassettes, as well as between the antibiotic cassette and the pUC19 vector. The cloning was performed with an insert to vector molar ratio of 3:3:1 (equimolar between both inserts) to compare the efficiency between the different HAC tail lengths, as well as between stopping with EDTA and repairing with dNTPs. By plating an equal volume of transformants on LB Amp plates and LB Cm/Kan plates (100 μl out of 1 ml recovered cells; 2.5 ng of vector), we were able to compare the success rate of inserting both cassettes into the vector based on the colonies that grew in the presence of both Cm and Kan (Table 2). As above, increasing the length of the HAC tails increased the total number of colonies obtained from the transformation, however when cloning two inserts together, it is evident that increased HAC primer tail length also improves efficiency with 40%, 71% and 79% positive clones achieved with 15, 20 and 25 bp HAC tails, respectively. According to this, we recommend the use of longer HAC tails for the cloning of multiple inserts. From our results, we would recommend a minimum of 20 bp as sufficient to obtain a high-level efficiency rate although it may be worthwhile to use up to 25 bp should more fragments be required to be cloned simultaneously. When comparing between the addition of dNTPs (gap repair) instead of EDTA, the addition of dNTPs increased the total number of colonies obtained, although again, not the efficiency based on the number of colonies on LB Cm/Kan. While we maintain that it is unnecessary to utilise dNTPs for the cloning of multiple inserts, should the competency of the cloning strain be an issue, utilising the alternative dNTPs method may improve the cloning process simply due to the increased number of colonies obtained.

**Table 2:** Efficiency of HAC for simultaneously cloning of two inserts.

Cloning of *cm^r^* and *kan^r^* into pUC19						
	Stopped with EDTA			Repaired with dNTPs		
	Kan/Cm colonies	Amp colonies	Approximate % success	Kan/Cm colonies	Amp colonies	Approximate % success
15 bp overhangs	38	96	40	124	296	42
20 bp overhangs	89	125	71	192	327	59
25 bp overhangs	189	239	79	351	558	63

### Optimised HAC method

Based on our efforts to optimise the HAC method, we present the following as the recommended HAC procedure:

1. Design primers for the amplification of desired insert while incorporating HAC tails of 15 bp for single insert and >20 bp for multiple inserts;
2. PCR amplify insert with HAC-tailed primers;
3. Clean up PCR product to remove unincorporated dNTPs with appropriate DNA clean-up kit;
4. Prepare the HAC reaction on ice, containing 50 ng of RE digested vector DNA, purified insert DNA (3:1 insert:vector molar ratio), T4 polymerase buffer and 1 U of T4;
5. Incubate at 37 °C for 1 min, then add EDTA to a final concentration of 25 nM and mix;
6. Incubate at 60 °C for 1 min and remove, allowing the reaction to cool to room temperature;
7. Transform into chemically competent *E. coli* via heat shock.

*Nb. Although not necessary, we routinely perform the HAC reaction in a thermocycler for convenience and accuracy.

### Comparison of HAC and one-step SLIC

In order to assert the benefits of our method amongst the different variations of LIC-based methods in the literature, we chose to perform a direct comparison of HAC with the one-step SLIC method (Islam et al., 2017; Jeong et al., 2012). The one-step SLIC was first described as an improvement and simplification of the original SLIC method, which while it was the earliest description of a LIC method without sequence restriction, it was also a somewhat complicated procedure (Li & Elledge, 2007). Among all the LIC-based methods, one-step SLIC is the most similar to HAC, but differs from HAC in two important ways; the first being that the T4 pol exonuclease activity is not inactivated, and the second, the annealing step relies on an extended period on ice. In order to directly compare these two important differences, we conducted HAC with the sub-optimal insert:vector molar ratio of 2:1 (as recommended in one-step SLIC) and with all other conditions identical. We cloned the *gfp* gene with 15 bp HAC-tails using one-step SLIC (50 °C for 30 s, 10 min on ice) (Islam et al., 2017) and HAC (37 °C for 1 min, EDTA, 1 min 60 °C). In our hands, one-step SLIC showed an efficiency of approximately 72% as compared to HAC at 92% efficiency (Fig. S1). It is worthwhile to note here that the efficiency of 92% achieved in this particular experiment is lower than that achieved when using our recommended insert:vector molar ratio for HAC of 3:1 (See above; Table 1).

## Discussion

The limitations of conventional ligase-based cloning serve as the main driving force behind the development of LIC-based methods. Despite the introduction of several LIC methods, many of these methods remain relatively obscure and have been unable to replace conventional ligation-based routine cloning work. HAC was developed in our laboratory as an easy and reliable way to perform routine cloning of PCR products. The aim of this manuscript is to illustrate clearly the benefits of this method, and how it can be easily adapted by anyone. Upon optimisation of the method, HAC is not only rapid and easy to perform, it boasts an incredible success rate, almost 100%, when cloning a single insert.

The T4 pol is essential to the process due to its 3′→5′ exonuclease activity. Indeed, T4 pol is also employed in other LIC methods including the use of the proprietary LIC plasmid pMCSG7 and its family of cloning vectors (Eschenfeldt et al., 2009; Stols et al., 2002). While the T4 pol is useful for generating 5′-extending single-stranded tails, it is necessary to stop the process to prevent complete degradation of the substrate. Some methods employ the usage of a single nucleoside triphosphate to halt the T4 pol exonuclease activity after a certain point (Aslanidis & de Jong, 1990; Stols et al., 2002); this, however, imposes a restriction on the templates suitable for the process. Instead, we chose to determine the optimal incubation time and condition that would allow the sufficient generation of single-stranded tails while minimising the risk of degrading even small products. The T4 pol exonuclease activity can then be effectively halted simply through the addition of EDTA at a final concentration of 25 mM. Subsequent removal of EDTA is not necessary and one can progress directly to transformation.

From our experiment utilising the Sybr Green I dye to quantify the decrease in double-stranded DNA of a single PCR product over a period of time, we have calculated the rate of T4 pol exonuclease activity to be approximately 100 bp/min. Previous estimates of the exonuclease rate of T4 pol have been reported as 40 bp/min (Rittie & Perbal, 2008). It is possible that in part, this discrepancy arises from improved enzyme preparations, buffer formulations and enzymes of increased activity. From our calculated rate, it appears that an incubating period of just 20 s would be sufficient to generate single-stranded tails that are long enough, and indeed this may well be true. However, our calculation is not meant to be precise and is based on several assumptions that are difficult to experimentally prove, one of which being that the T4 pol exonuclease activity rate would be equal across all substrates. Our selection of an incubation time of 1 min at 37 °C remains our conservative recommended time that would ensure sufficient generation of single-stranded tails, even with less efficient enzymes, while not completely degrading smaller products of down to 200 bp, as evidenced in Fig. 2B. From these results, we would recommend that for the cloning of very short fragments (<200 bp), further reduction of exonuclease duration can be used to preserve high cloning efficiencies.

In this demonstration of HAC, we have deliberately been conservative in our approach to illustrate the utility of this method for the greatest number of researchers. In doing so we have considered several method parameters which may vary across laboratories and have thus reported the conditions which we consider easily replicated. We have not attempted to define cloning efficiency in terms of recombinant vector formation frequency, or transformation success per quantity of DNA; indeed, we have deliberately utilised cells of modest competency. When reporting the efficiency of HAC, we do so to define the percentage of resulting transformants, positive for the desired recombinant vector, i.e. percentage of transformants with the desired insert cloned. We believe this information will benefit the majority of researchers attempting to clone PCR products, but do not discount that some will have a particular interest in the rate of insert integration and subsequent yield of transformants. While this is outside the scope of the current study, we have shown that the use of longer HAC tails will yield a greater number of colonies overall if desired (Table 1).

We were interested to determine if halting exonuclease activity through the addition of dNTPs, which would also result in the repair of single-stranded gaps on the annealed insert-vector, may improve the method, potentially by way of greater vector stability. Interestingly, when cloning a single insert, not only did the use of dNTPs not improve the method, we actually saw a decrease in both numbers and efficiency (Table 1). A possible reason may be that upon the subsequent repairing of all single-stranded molecules with dNTPs, any further annealing between the insert and vector prior to transformation is inhibited. This is in contrast to stopping the procedure with EDTA, where assessable fragment ends are present throughout the final stages of the procedure. This possibility reinforces the benefit of utilising EDTA to quench T4 pol activity prior to annealing, as per the HAC protocol.

When cloning two inserts, the use of dNTPs likewise did not result in an increase in efficiency, however we did observe an increase in the total number of colonies obtained, possibly due to greater plasmid stability through single-stranded gap repair. While we maintain that it is unnecessary to utilise dNTPs for the cloning of multiple inserts, should the competency of the cloning strain be an issue, utilising the alternative dNTPs method may improve the cloning process simply due to the increased number of colonies obtained. Although in saying that, it is important to highlight that the modest level of competency of the cells used in this study (5 × 10^6^ cfu/μg of pUC19) indicates that highly competent cells are not necessary for routine cloning, further saving costs.

We have provided above a detailed analysis of our method by cloning genes with easily measurable phenotypes, as well as a detailed description of how to perform it, including the procedure for HAC primer design. Although in principle, the concept of the one-step SLIC method (Islam et al., 2017; Jeong et al., 2012) is most analogous to HAC of all the other LIC-based methods, we present evidence that in our hands, HAC remains a highly reliable method that balances both ease of use, speed and boasting higher levels of cloning efficiencies. We attribute our increased cloning success rates to the effective termination of T4 pol exonuclease activity, together with the utilisation of a pre-annealing, 1 min 60 °C incubation, to increase the rates and specificity of annealing between the insert and vector homology sequences, as demonstrated in Fig. S1.

## Conclusion

In conclusion, we have demonstrated that HAC is a fast, easy and highly efficient cloning method that has the potential to replace the use of conventional cloning in any laboratory for most purposes. While our results boast a near 100% cloning efficiency when cloning a single PCR product into pUC19, it is important to qualify that this is ‘demonstrative’ in nature and as such, the exact efficiencies may vary when cloning different inserts/vectors. However, since the development of the HAC protocol, our laboratory has successfully produced scores of clones, with inserts from various origins, length, complexity and GC content, cloned into many different plasmids. The incredible cloning success rates together with the multiple practical benefits of low cost, vector flexibility, insert orientation and speed highlights the potential of HAC to replace conventional ligase-dependent cloning for most routine molecular cloning work.

## Supplemental Information

10.7717/peerj.5146/supp-1 Supplemental Information 1 Table S1. Primers used and plasmids constructed in this study.

10.7717/peerj.5146/supp-2 Supplemental Information 2 Fig. S1. Assessment of principle methodological differences between HAC and one-step SLIC. The *gfp*gene with 15 bp HAC tails was cloned into pUC19 via HAC or one-step SLIC at the SLIC recommended insert:vector molar ratio of 2:1 (sub-optimal ratio for HAC). 100 μl of each transformation was plated on LB agar supplemented with Ampicillin. Assessment of colonies after 18 hr growth was performed with a photographic imager equipped with fluorescence detection. Images depicted were captured in fluorescent (Cy3 filter) + colorimetric overlay camera mode. Fluorescent colonies are coloured green. Total numbers of fluorescing colonies as a percentage of total CFUs represent 92% for HAC and 72% for SLIC.

10.7717/peerj.5146/supp-3 Supplemental Information 3 Raw data.

## Additional Information and Declarations

### Data Availability

The following information was supplied regarding data availability: The raw data are provided in a Supplemental File.
