# Supplementary material for: Homologous alignment cloning: a rapid, flexible and highly efficient general molecular cloning method
Source: PeerJ. 2018 Jun 29;6:e5146. doi: 10.7717/peerj.5146 (PMC6054264; doi:10.7717/peerj.5146)
Supplement: Supplemental Information 1 [file peerj-06-5146-s001.docx]

| **Primers used in this study** | | |
| --- | --- | --- |
| **Primer sequence** | | **Description** |
| TGATTACGCCAAGCT GAAGGAGATATACATATGAG | | Forward primer to amplify *gfp* with 15 bp HAC tails to clone into pUC19 at HindIII site |
| GCAGGCATGCAAGCT TTATTTGTATAGTTCATCCATGC | | Reverse primer to amplify *gfp* with 15 bp HAC tails to clone into pUC19 at HindIII site |
| GACCATGATTACGCCAAGCT GAAGGAGATATACATATGAG | | Forward primer to amplify *gfp* with 20 bp HAC tails to clone into pUC19 at HindIII site |
| GACCTGCAGGCATGCAAGCT TTATTTGTATAGTTCATCCATGC | | Reverse primer to amplify *gfp* with 20 bp HAC tails to clone into pUC19 at HindIII site |
| GCTATGACCATGATTACGCCAAGCT GAAGGAGATATACATATGAG | | Forward primer to amplify *gfp* with 25 bp HAC tails to clone into pUC19 at HindIII site |
| GAGTCGACCTGCAGGCATGCAAGCT TTATTTGTATAGTTCATCCATGC | | Reverse primer to amplify *gfp* with 25 bp HAC tails to clone into pUC19 at HindIII site |
| TGATTACGCCAAGCT TCCTGGTGTCCCTGTTGATAC | | Forward primer to amplify *cm^r^* with 15 bp HAC tails to clone into pUC19 along with *kan^r^* at HindIII site |
| CGTAGGTACTGGTAC ACACGTCTTGAGCGATTGTG | | Reverse primer to amplify *cm^r^* with 15 bp HAC tails to clone into pUC19 along with *kan^r^* at HindIII site |
| GTACCAGTACCTACG GAAAGCCAGTCCGCAGAAAC | | Forward primer to amplify *kan^r^* with 15 bp HAC tails to clone into pUC19 along with *cm^r^* at HindIII site |
| GCAGGCATGCAAGCT GGCGGTGGAATCGAAATCTC | | Reverse primer to amplify *kan^r^* with 15 bp HAC tails to clone into pUC19 along with *cm^r^* at HindIII site |
| GACCATGATTACGCCAAGCT TCCTGGTGTCCCTGTTGATAC | | Forward primer to amplify *cm^r^* with 20 bp HAC tails to clone into pUC19 along with *kan^r^* at HindIII site |
| GATGTCGTAGGTACTGGTAC ACACGTCTTGAGCGATTGTG | | Reverse primer to amplify *cm^r^* with 20 bp HAC tails to clone into pUC19 along with *kan^r^* at HindIII site |
| GTACCAGTACCTACGACATC GAAAGCCAGTCCGCAGAAAC | | Forward primer to amplify *kan^r^* with 20 bp HAC tails to clone into pUC19 along with *cm^r^* at HindIII site |
| GACCTGCAGGCATGCAAGCT GGCGGTGGAATCGAAATCTC | | Reverse primer to amplify *kan^r^* with 20 bp HAC tails to clone into pUC19 along with  *cm^r^* at HindIII site |
| GCTATGACCATGATTACGCCAAGCT TCCTGGTGTCCCTGTTGATAC | | Forward primer to amplify *cm^r^* with 25 bp HAC tails to clone into pUC19 along with *kan^r^* at HindIII site |
| CTTCAGATGTCGTAGGTACTGGTAC ACACGTCTTGAGCGATTGTG | | Reverse primer to amplify *cm^r^* with 25 bp HAC tails to clone into pUC19 along with *kan^r^* at HindIII site |
| GTACCAGTACCTACGACATCTGAAG GAAAGCCAGTCCGCAGAAAC | | Forward primer to amplify *kan^r^* with 25 bp HAC tails to clone into pUC19 along with *cm^r^* at HindIII site |
| GAGTCGACCTGCAGGCATGCAAGCT GGCGGTGGAATCGAAATCTC | | Reverse primer to amplify *kan^r^* with 25 bp HAC tails to clone into pUC19 along with  *cm^r^* at HindIII site |
| **Plasmids constructed in this study** | | |
| **Name** | **Description** | |
| pUC19:*gfp*15 | *gfp* gene cloned into HindIII site of pUC19 via 15 bp HAC tails | |
| pUC19:*gfp*20 | *gfp* gene cloned into HindIII site of pUC19 via 20 bp HAC tails | |
| pUC19:*gfp*25 | *gfp* gene cloned into HindIII site of pUC19 via 25 bp HAC tails | |
| pUC19:*cm-kan*15 | *cm^r^* and *kan^r^* resistance cassettes cloned into HindIII site of pUC19 via 15 bp HAC tails | |
| pUC19:*cm-kan*20 | *cm^r^* and *kan^r^* resistance cassettes cloned into HindIII site of pUC19 via 20 bp HAC tails | |
| pUC19:*cm-kan*25 | *cm^r^* and *kan^r^* resistance cassettes cloned into HindIII site of pUC19 via 25 bp HAC tails | |
