# Supplementary figures and images for: Homologous alignment cloning: a rapid, flexible and highly efficient general molecular cloning method
Source: PeerJ. 2018 Jun 29;6:e5146. doi: 10.7717/peerj.5146 (PMC6054264; doi:10.7717/peerj.5146)

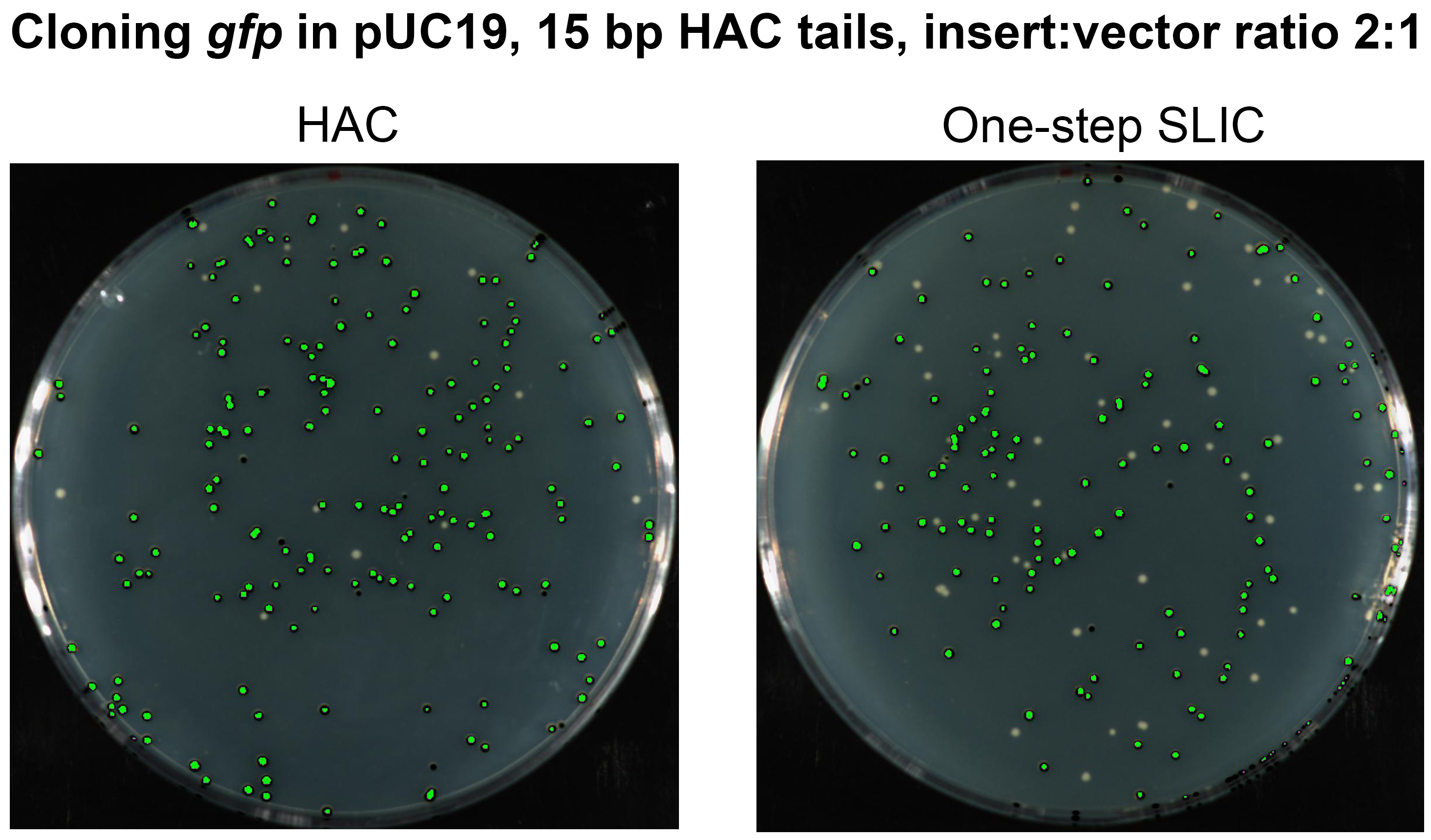

Supplement: Supplemental Information 2 — The gfpgene with 15 bp HAC tails was cloned into pUC19 via HAC or one-step SLIC at the SLIC recommended insert:vector molar ratio of 2:1 (sub-optimal ratio for HAC). 100 μl of each transformation was plated on LB agar supplemented with Ampicillin. Assessment of colonies after 18 hr growth was performed with a photographic imager equipped with fluorescence detection. Images depicted were captured in fluorescent (Cy3 filter) + colorimetric overlay camera mode. Fluorescent colonies are coloured green. Total numbers of fluorescing colonies as a percentage of total CFUs represent 92% for HAC and 72% for SLIC. [file peerj-06-5146-s002.png]
