# Supplementary material for: Homologous alignment cloning: a rapid, flexible and highly efficient general molecular cloning method
Source: PeerJ. 2018 Jun 29;6:e5146. doi: 10.7717/peerj.5146 (PMC6054264; doi:10.7717/peerj.5146)
Supplement: Supplemental Information 3 [file peerj-06-5146-s003.docx]

**Raw data**

**Figure 2A plot points**

| **Time (seconds)** | **Relative fluorescence unit** |
| --- | --- |
| 0 | 91845 |
| 18 | 91200 |
| 36 | 85023 |
| 54 | 80945 |
| 72 | 74452 |
| 90 | 69853 |
| 108 | 60625 |
| 126 | 62140 |
| 144 | 56106 |
| 162 | 46653 |
| 180 | 41078 |
| 198 | 36027 |
| 216 | 33674 |
| 234 | 26761 |
| 252 | 27048 |
| 270 | 24735 |
| 289 | 22445 |
| 308 | 21476 |
| 327 | 19150 |
| 346 | 19618 |
| 365 | 19758 |
| 384 | 21284 |
| 402 | 19027 |
| 420 | 18115 |
| 480 | 18209 |
| 600 | 17126 |
| 720 | 16087 |
| 840 | 17126 |
| 960 | 16296 |
| 1080 | 16836 |

**Figure 3 colony counts (replicated from Table 1)**

| **Cloning of *gfp* into pUC19** | | | | | | |
| --- | --- | --- | --- | --- | --- | --- |
|  | **Stopped with EDTA** | | | **Repaired with dNTPs** | | |
|  | **Fluorescent colonies** | **Total colonies** | **% success** | **Fluorescent colonies** | **Total colonies** | **% success** |
| **15bp overhangs** | 217 | 226 | 96 | 143 | 175 | 82 |
| **20 bp overhangs** | 458 | 467 | 98 | 367 | 415 | 88 |
| **25 bp overhangs** | 754 | 761 | 99 | 625 | 686 | 91 |

**Figure S1 colony counts**

| **Cloning of *gfp* into pUC19 at 2:1 insert:vetor molar ratio** | | | | | |
| --- | --- | --- | --- | --- | --- |
| **HAC** | | | **One-step SLIC** | | |
| **Fluorescent colonies** | **Total colonies** | **% success** | **Fluorescent colonies** | **Total colonies** | **% success** |
| 174 | 188 | 92 | 134 | 187 | 72 |
